# Effects of pathogen dependency in a multi-pathogen infectious disease system including population level heterogeneity – a simulation study

**DOI:** 10.1186/s12976-017-0072-7

**Published:** 2017-12-13

**Authors:** Abhishek Bakuli, Frank Klawonn, André Karch, Rafael Mikolajczyk

**Affiliations:** 1 Helmholtz Centre for Infection Research, Research Group Biostatistics, Braunschweig, Germany; 2 PhD Programme “Epidemiology”, Braunschweig-Hannover, Germany; 3 Department of Computer Science, Ostfalia University of Applied Sciences, Wolfenbuettel, Germany; 4 Helmholtz Centre for Infection Research, Department of Epidemiology, Braunschweig, Germany; 5 Hannover Medical School, Hannover, Germany; 6 Institute for Medical Epidemiology, Biometry, and Informatics (IMEBI), Medical Faculty of the Martin Luther University Halle-Wittenberg, Halle (Saale), Germany

**Keywords:** Agent based model, Epidemic, Household size, Pathogen dependency, Multi-pathogen

## Abstract

**Background:**

Increased computational resources have made individual based models popular for modelling epidemics. They have the advantage of incorporating heterogeneous features, including realistic population structures (like e.g. households). Existing stochastic simulation studies of epidemics, however, have been developed mainly for incorporating single pathogen scenarios although the effect of different pathogens might directly or indirectly (e.g. via contact reductions) effect the spread of each pathogen. The goal of this work was to simulate a stochastic agent based system incorporating the effect of multiple pathogens, accounting for the household based transmission process and the dependency among pathogens.

**Methods:**

With the help of simulations from such a system, we observed the behaviour of the epidemics in different scenarios. The scenarios included different household size distributions, dependency versus independency of pathogens, and also the degree of dependency expressed through household isolation during symptomatic phase of individuals. Generalized additive models were used to model the association between the epidemiological parameters of interest on the variation in the parameter values from the simulation data. All the simulations and statistical analyses were performed using R 3.4.0.

**Results:**

We demonstrated the importance of considering pathogen dependency using two pathogens, and showing the difference when considered independent versus dependent. Additionally for the general scenario with more pathogens, the assumption of dependency among pathogens and the household size distribution in the population cohort was found to be effective in containing the epidemic process. Additionally, populations with larger household sizes reached the epidemic peak faster than societies with smaller household sizes but dependencies among pathogens did not affect this outcome significantly. Larger households had more infections in all population cohort examples considered in our simulations. Increase in household isolation coefficient for pathogen dependency also could control the epidemic process.

**Conclusion:**

Presence of multiple pathogens and their interaction can impact the behaviour of an epidemic across cohorts with different household size distributions. Future household cohort studies identifying multiple pathogens will provide useful data to verify the interaction processes in such an infectious disease system.

**Electronic supplementary material:**

The online version of this article (10.1186/s12976-017-0072-7) contains supplementary material, which is available to authorized users.

## Background

Respiratory infections are the most common type of infections that contribute to loss of productive time due to acute conditions [1]. Households play an important role for the transmission process of respiratory infective agents, since they serve as confined structures due to the proximity of contacts among individuals that belong to such a confinement [2]. Approximately a third of the influenza like infection transmissions occur within households [3–5]. Studies on modelling epidemics spread in populations distributed into household clusters of varying sizes have been conducted to investigate possible control measures against epidemic outbreaks where larger households were associated with more infection transmissions [6–10].

Individual level stochastic models, also known as agent based models are highly flexible constructs to study complex phenomena by simulating the behaviour of multiple agents (individuals or grouped entities) simultaneously. FluTE [11] and FRED [12] are examples of such agent based models that have been built incorporating the community structure to study the progression of influenza like infections in the population [13–15].

Epidemic studies, till date, have mostly focused on the effect of a single pathogen in determining the population behaviour and spread of infections. Seasonal epidemics of respiratory infections are a common phenomenon during the winter months annually with several emergent and dominant pathogens circulating in the society. Additionally, there is always the possibility for antigenic drifts which are due to mutations of viruses impacting the protective effect of immunity from further infections [16]. Thus there is a need to study the epidemic reality of several pathogens co-existing in the community, with differential seasonality patterns, as well as differential severity and transmissibility characteristics. The idea of dynamic interaction between pathogens or ecological interference has been studied for diseases with differential seasonality in case of measles and whopping cough [17] and for the impact of vaccination for pandemic influenza [18].

The study of the infection process with multiple interacting pathogens has been lacking in the agent based models that have been developed in the past. Infection from one pathogen along with an intervention strategy, like household isolation, can not only have an impact on the individual’s exposure to the specific pathogen but also to other pathogens which can eventually impact parallel epidemic processes from other co-existing pathogens. This involves cross immunity caused by an infectious pathogen, and changes in the contact structure among individuals within and between households. In addition to this, if there are two pathogens with exactly the same characteristics, they create a competition within the scope of the epidemic process. Additional factors like household structure and presence of an immunized proportion of individuals can impact the course of the epidemic since they can potentially accelerate or decelerate the transmission of infections in the population [8–10]. Moreover they are also directly related to the household isolation strategy since they impact the within household transmission. The aim of our study is to investigate how multi-pathogen interaction impacts the epidemic process when compared to scenarios with only a single pathogen if different household structures and the proportion of already immune individuals are taken into account.

## Methods

### Agent-based modelling of disease transmission

We use an agent-based approach with the basic structure of an SEIR (Susceptible, Exposed, Infectious, and Recovered) model. During the exposed state we assume that individuals are asymptomatic and do not impact the transmission process. After a period of being asymptomatic the individuals enter the infectious phase where they are symptomatic and can transmit infections. The assumption that during the infectious phase, there is household isolation making an individual nullify the risk of external infection from other pathogens, causes the interaction between pathogens in the multi-pathogen setting. The degree of this reduction of the external transmissibility depends on pathogen characteristics.

The single pathogen case is shown in Table 1. *TP*_*1*_ *(t)* has two components: transmission of the pathogen resulting from contacts in the society (*P*_*external*_(*p*, *t*)) and from contacts within households ( *P*_*family*_(*p*, *t*)) for a given pathogen *p*. Transmission in the society depends on baseline infectivity of the pathogen (*v*), proportion of infectious in the society reduced by pathogen specific factor *z*, and a seasonality parameter (*s*(*t*)).

**Table 1:** The transition probability matrix for a single pathogen with the SEIR states

			Time = t + 1		
		Susceptible	Exposed	Infectious	Recovered
	Susceptible	1 − *TP*_1_(*t*)	*TP*_1_(*t*)	0	0
Time = t	Exposed	0	1 − *TP*_2_	*TP*_2_	0
	Infectious	0	0	1 − *TP*_3_	*TP*_3_
	Recovered	0	0	0	1

$$ {P}_{external}\left(p,t\right)=v\ s(t)\ z\ \left(\frac{I\left(t-1\right)}{N}+{P}_0\right), $$with $$ N=S(t)+E(t)+I(t)+R(t)\  and\ \frac{I\left(t-1\right)}{N}+{P}_0\le 1 $$.

*s*(*t*) = *A* (sin (*ω* (*t* − *t*_0_)) + 1)/2, *ω* = 2*π*/365, (if simulation starts on October 1st then *t* = 0, *t*_0_ = 0). *P*_0_ indicates external influx of infection, *A* indicates the amplitude of the seasonality function (for example the effects of outside temperature, Table 2). The parameters are calibrated to restrict *P*_*external*_(*t*) with the upper limit as one.

**Table 2:** Description of the symbols used in the mathematical formulation of the transition probabilities for describing the agent based model

Symbols	Description
*N*	Total number of individuals in the cohort (10,000 individuals considered as a population cohort)
*S*(*t*); *E*(*t*); *I*(*t*); *R*(*t*)	Number of individuals in the Susceptible, Exposed, Infectious, and Recovered states at time point *t*
*P*_*external*_(*t*)	Probability of a susceptible individual acquiring infections from contacts in society
*P*_*family*_(*t*)	Probability of a susceptible individual acquiring infections from contacts within household
*v*	Baseline infectivity of a given pathogen. Always present in determining the probability of acquiring an infection by a susceptible individual (Fixed at 0.025 for each day)
(*I*(*t*))/*N*	Proportion of infectious individuals in society at time *t.* Impacts the probability of susceptible individuals acquiring infections from society at time *t + 1*
*P*_0_	Influx of infection from outside of the studied population to avoid permanent extinction of the epidemics (fixed at 0.0001 for a single day)
*z*	Pathogen specific reduction factor; Expression of severity of symptoms thus extent of isolation from the society; Multiplicative factor on the sum of the proportion of infectious individuals and the influx of infections from outside the population (Range: 0.3–0.9)
*s*(*t*)	Seasonality parameter at time point *t*; expression for the seasonal variability in the transmission probability of the infection from contacts at the society level for a specific pathogen
*A*	Amplitude for the seasonality characteristics of the pathogen; indicates the extent of seasonal variation of transmissibility of a given pathogen (Range: 0.5–5, lower values indicate lack of seasonality whereas higher values are indicative of seasonality)
*c*	Factor for increased closeness of contacts within household as differentiated from the society contacts; Multiplicative factor on the baseline infectivity for determining the within household transmission probability for a specific pathogen (Fixed at 9 for all pathogens)
Λ	Coefficient for the degree of household isolation. In case of complete pathogen dependency with full household isolation of 100%, risk of acquiring infections from outside household when already infectious is zero. For the independent pathogens scenario, the household isolation is 0% which means that there is a complete risk of acquiring a co-infection from outside household.
*t*_0_	Coefficient for the phase shift. It helps in varying the temporal trend of the pathogen. It is set to zero for most cases. Except for pathogen 10, we examine the case when the value is +/− 45 days and remains zero for the other pathogens
*I*_*h*_(*t*)	Number of infectious persons in the same household at time *t*; impacts the probability of acquiring an infection from household contacts at time *t + 1*; exponential factor on the product of baseline infectivity and closeness of contacts
*LP*	Length of asymptomatic infection(latency period);Average time spent from being exposed to becoming infectious for a specific pathogen (Range: 1–6)
*IP*	Length of symptomatic infections(infectious period); Average time spent in-between becoming infectious and acquiring immunity (Range: 2–9)

Transmission in the family depends on pathogen characteristics - baseline infectivity *v*, factor for within family closeness of contacts  *c* and number of infectious persons in the same household *I*_*h*_(*t*).

${P}_{family}\left(p,t\right)=1-{\left(1-v\ c\right)}^{I_h\left(t-1\right)}$ (Description provided in Table 2.)

*Z*(*t*) ∈ {*Susceptible*, *Exposed*, *Infectious*, *Recovered*} ∀ *t* ≥ 1, where Z is an individual in the study.

*TP*_1_ = *Probability*(*Z*(*t* + 1) = *Exposed* | *Z*(*t*) = *Susceptible*) (Table 1).

=1 − (1 − *P*_*external*_(*p*, *t*)) ∗ (1 − *P*_*family*_(*p*, *t*)) (Table 2).

The probability *TP*_1_ describes the transition probability from being S*usceptible* to becoming *Exposed*. The above formulation includes the specific scenario, when there is no possibility of a family based transmission, which is always the case for a single member household.

Let LP (>0) and IP (>0) (description in Table 2) be the average latency period and infectious period, respectively for a given pathogen *p. TP*_*2*_ describes the transition probability of an *Exposed* individual becoming *Infectious* for the pathogen it is already exposed to. *TP*_*3*_ describes the transition probability for an *Infectious* individual to obtain immunity or become *Recovered* for that pathogen for the remaining time in the study period. In this paper, we assume that *LP* and *IP* are independent constructs.

*TP*_2_ = *Probability*(*Z*(*t* + 1) = *Infectious* | *Z*(*t*) = *Exposed*) (Table 1)

$$ \min \left(\frac{1}{LP},1\right)=q={TP}_2 $$

*X*(*i*, *p*)~*Geometric*(*q*)− After time  *X*(*i*, *p*), that is, at time *X*(*i*, *p*) + 1, the *i* ^*th*^ individual becomes *Infectious,* since the time it became *Exposed* for pathogen *p*. (Description in Table 2).

*TP*_3_ = *Probability*(*Z*(*t* + 1) = *Recovered* | *Z*(*t*) = *Infectious*) (Table 1)

$$ \min \left(\frac{1}{IP},1\right)=r={TP}_3 $$

*Y*(*i*, *p*)~*Geometric*(*r*)− After time *Y*(*i*, *p*), that is, at time *Y*(*i*, *p*) + 1 the *i* ^*th*^ individual acquires immunity, since the time it became *Infectious* from the pathogen *p,* for the remaining study period (Description in Table 2).We also assume that *X*(*i*, *p*) and *Y*(*i*, *p*) are independently distributed as a geometric distribution.

**Fig. 1:**
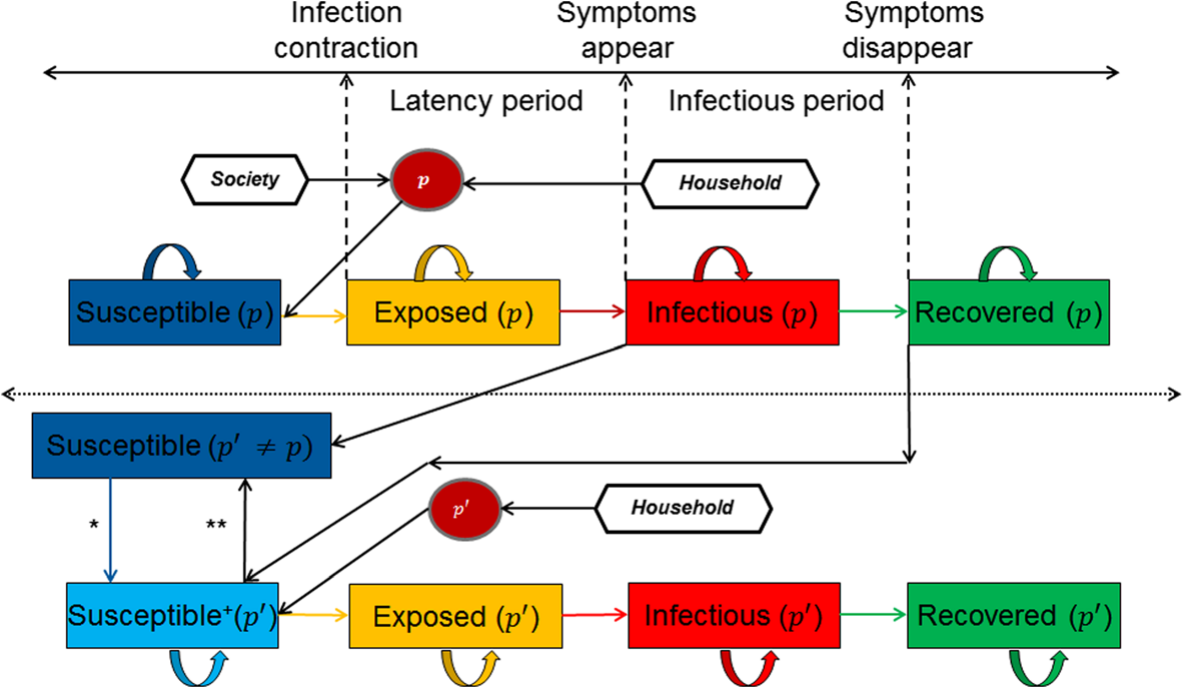
Graphical illustration of Susceptible, Exposed, Infectious, and Recovered states of the agent based model with some assumptions described. The time lines for the latency period and infectious period are also indicated through the dashed lines for an i^th^ individual in the population for pathogens p and p^'^, where p^'^ ≠ p. The dependency assumption induces the *Susceptible* ^*+*^ state. The black arrows represent the influence direction, whereas the coloured arrows represent the transitions. The part above the dotted line indicates the states when only one pathogen is present in society, or when the pathogens are independently functioning in the system. The part below the dotted line is introduced when more than one pathogen is present in society and the pathogens interfere in the joint behaviour. *When an individual is *Infectious* for pathogen p and is still susceptible for another pathogen *p* ^'^ it instantaneously moves to the state *Susceptible* ^*+*^ for pathogen *p* ^'^ .** Once the individual is at the *recovered* state for pathogen *p* and is still at state *Susceptible* ^*+*^ for pathogen *p* ^'^ it switches back to *Susceptible* state instantaneously

**Fig. 2:**
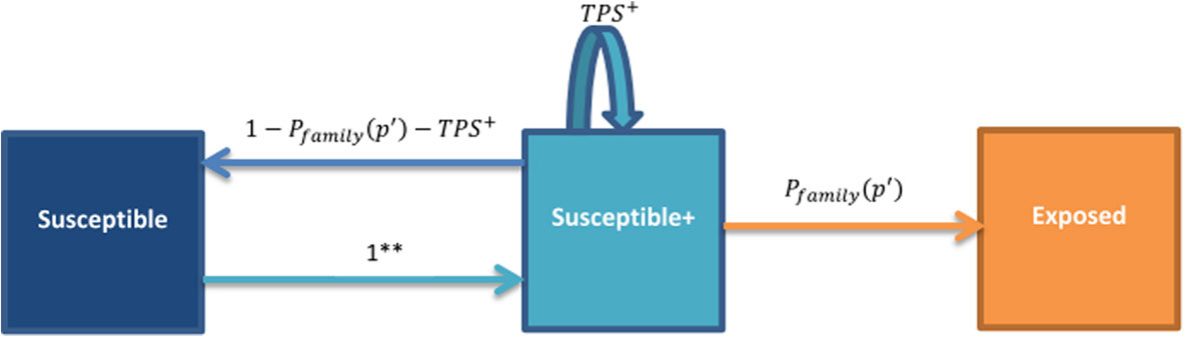
Markov chain describing the dependency process among pathogens. ** Once the individual is in the *Recovered state* for pathogen *p* and is still at *Susceptible* ^*+*^ state for pathogen *p* ^'^ it switches back to *Susceptible* state instantaneously

When there are multiple pathogens present in the society (*p and p* ^'^ in our case are two different exemplary pathogens), we introduce an additional state of *Susceptible* ^*+*^ in the agent based model. On acquiring symptoms of infection (i.e. state as *Infectious*) with pathogen *p*, there is a check to verify if an individual is susceptible for another pathogen’*,* i.e. *Susceptible(p* ^'^ *)* is True or False. If True, then the individual at *Susceptible (p* ^'^ *)* moves to *Susceptible* ^*+*^ *( p* ^'^ *)* instantaneously. Once an individual at *Infectious(p)* moves to *Recovered(p),* we check once again if the individual is still susceptible to *p* ^'^ *,* i.e. *Susceptible* ^*+*^ *( p* ^'^ *)* is True or False. If True then *Susceptible* ^*+*^ *( p* ^'^ *)* moves to *Susceptible(p* ^'^ *)* instantaneously (Fig. 1). A person at the state of *Susceptible* ^*+*^ is potentially at risk only to the household mode of infection transmission and can become *Exposed*. Following this, the steps for the exposed individual are the same as described for one pathogen. In case the individual reaches the state *Recovered* for *p*, while it is still at *Susceptible* ^*+*^ for some *p* ^'^, then it becomes *Susceptible* once again for *p* ^'^. The described process is pictorially represented through a Markov chain (Fig. 2). We vary the degree of household isolation using a parameter λ which takes values between zero and one to indicate differences in the risk of acquiring an infection from outside the household.

$$ Probability\left(\boldsymbol{Z}\left(\boldsymbol{t}+1\right)={Susceptible}^{+}\kern0.1em |\kern0.1em \boldsymbol{Z}\left(\boldsymbol{t}\right)= Susceptible\right)=1\kern0.1em \mathrm{for}\kern0.17em \mathrm{pathogen}\kern0.1em {p}^{\hbox{'}} $$ (Fig. 1).

When *Z*(*t*) = *Infectious* for pathogen *p* and *p* ≠ *p* ^'^.

The description of *P*_*family*_ remains unchanged. *TPS* ^+^ describes the probability that, an individual at the state *Susceptible* ^+^ remains as *Susceptible* ^+^. This indicates the situation where the individual does not get exposed to a pathogen *p* ^'^ but remains symptomatic for *p*. This depends on the pathogen characteristics for both *p and p* ^'^ .

$$ {TPS}^{+}= Probability\left(\boldsymbol{Z}\left(\boldsymbol{t}+1\right)={Susceptible}^{+}\kern0.5em |\ \boldsymbol{Z}\left(\boldsymbol{t}\right)={Susceptible}^{+}\right) $$

=(1 − (1 − *P*_*family*_(*p* ^'^, *t*)) ∗ (1 − (1 − *λ*)*P*_*external*_(*p* ^'^, *t*))) ∗ (1 − *TP*_3_( *p*)) (Fig. 2).

## Simulation

### Population structure

We have considered three population structures with different properties (Germany, India, one-person structure). The data on the household size distribution in Germany was used from DESTATIS (Statistisches Bundesamt, Wiesbaden 2015 report) while that of India from the census reports of 2011 [19, 20]. We have also considered a hypothetical population of one person households as the most extreme scenario. This has been described with the frequencies for each household size in Table 3. We distributed 10,000 individuals into each population scenario.

**Table 3:** The household size distributions for the different populations considered to describe the epidemic outcomes from simulations using the agent based model

Household size	1	2	3	4	5	6	7	8	9
Germany [19]	41.0%	34.0%	12%	7.5%	4.5%	1.0%	0%	0%	0%
India [20]	3.9%	8.2%	14.0%	16.9%	17.0%	15.2%	14.2%	8.2%	2.4%
Hypothetical	100%	0%	0%	0%	0%	0%	0%	0%	0%

### Pathogen characteristics

We studied a general multi-pathogen setting with n (*n* = 10) pathogens with characteristics chosen to reflect potential real life situations as described in Table 4. Baseline infectivity *v* was calibrated to achieve a maximum incidence rate of approximate 10% in person weeks for respiratory infections during the peak winter season (https://grippeweb.rki.de). Two broad types of pathogens were considered, the influenza type and the common cold type. Influenza type pathogens have typically reported shorter latent periods (period with asymptomatic infection; 1–4 days for influenza and common cold between 1 and 6 days) but a longer infectious period (period with symptomatic infection; 5–9 days for Influenza and 2–3 days for Common cold) while it has been reported as the opposite for common cold type of pathogens [21–23].

**Table 4:** Pathogen characteristics. This table with the input parameters for the simulation of the agent based model with ten pathogens. *I* indicates influenza type while *C* indicates common cold type of pathogen

Pathogen Characteristics		Pathogen Number									
Interpretation	***Symbol***	***1***	***2***	***3***	***4***	***5***	***6***	***7***	***8***	***9***	***10***
Seasonality	A	0.5	0.5	3.0	1.5	2.0	4.0	1.0	3.0	2.5	5.0
Baseline infectivity	*v*	0.025	0.025	0.025	0.025	0.025	0.025	0.025	0.025	0.025	0.025
Closeness family to society	*c*	9	9	9	9	9	9	9	9	9	9
Reduction of contacts with society	*z*	0.6	0.9	0.3	0.4	0.4	0.8	0.7	0.3	0.6	0.9
Duration of latent period(days)	*X*	1.5	3.0	6.0	5.0	4.0	4.0	2.0	4.0	1.5	1.5
Duration of infectious period(days)	*Y*	4	4	7	3	5	9	3	6	3	4
Proportion immune at start	*R*(1)	0.50	0.20	0.20	0.25	0.50	0.15	0.30	0.20	0.20	0.15
Number infectious at start(per 10,000)	*I*(1)	17	18	71	62	66	52	45	58	06	52
Number of infections from outside (per 10,000)	*P*_0_	**1**	1	1	1	1	1	**1**	1	1	1
Pathogen type		I	C	C	C	C	I	I	C	I	C

### Computation

The simulation proceeded in discrete time steps. Each step denoted a day in the follow up period. Based on the initial number of *Infectious* individuals, the epidemic process began its course of action. It followed the seasonal trend of the pathogen, the relation to other household members, and the prevalence of the infection for the specific pathogen in society at a given time point. We started initially with two pathogens from Table 4 (Pathogen 6 and Pathogen 10). Pathogen 6 would be in accordance with the characteristics of a pandemic influenza strain whereas pathogen 10 would correspond to the characteristics of human respiratory syncytial virus (HRSV). Then we observed the scenario where both the pathogens jointly interact. Finally we looked at the general scenario with 10 pathogens jointly which would be a more appropriate representation of the reality during the winter season [18] (https://grippeweb.rki.de).

The comparisons were done for the scenarios of pathogen dependencies, 1) assuming all pathogens existed independently (λ = 0%), and 2) assuming the pathogens worked together and influenced each other (λ = 100%) (indicated by Pathogen Dependency- Yes or No); household size distribution based on different household size distributions in different countries (Country – Germany, India or Hypothetical). At the start of the simulation few people were infectious for every pathogen, which was denoted as *I*(1) to kick start the infection process, while the number of people already immune at the start were represented as *R*(1).

In addition to the above, we assumed that, for every pathogen there would be a small chance that an individual could acquire an infection from outside the system. This has been described as the external influx of infection. We had set this value to one in a ten thousand, at each observational time point (day) in the epidemic process. Besides this, the maximum number of days spent as infectious had been censored to 55 days. Each of the scenario combinations were replicated 100 times for a study period of 150 days in the peak season for respiratory illness.

In our base case scenario with the German population and pathogen dependency (λ = 100%), we look at the same temporal tend (seasonality) for all the pathogens considered. However to present the effect of differential seasonality, we introduce a different temporal trend for pathogen 10, by modifying the value of *t*_0_ as +45 days and −45 days. This results in shifting the peak of the epidemic for these pathogens and impacts the overall epidemic process when multiple pathogens are present. Also for this scenario we evaluated the effect of change in λ from 0% to 100% in steps of 10% which would allow us to infer on the importance of the pathogen dependency assumption through the introduction of household isolation.

### Statistical methods

We measure the epidemiological parameters of interest which are 1) height of the epidemic peak (peak prevalence), 2) time taken to reach the peak of the epidemic, 3) incidence proportion (attack rate) of infections in the study period, and 4) incidence proportion stratified by household size for the different populations in consideration, through our simulations as described above. Summary statistics are presented for all the outcomes described above.

We observe the peak prevalence and the incidence proportion for the pathogens 6 and 10, both individually and jointly. We are interested in the hypothesis that jointly modelling pathogens creates a competition, and hence we would observe lower values of the peak prevalence and incidence proportion, compared to observing them individually. The observations are compared using the non-parametric Mann–Whitney–Wilcoxon test for evaluating the difference when observing joint epidemics. The parametric version with the paired t-test also gives us similar results, however due to no necessity of normal distribution assumptions the Mann–Whitney–Wilcoxon test values are reported [24].

We also use a simple linear regression model [25] on the outcomes described above and show the confidence intervals for the slope across different outcomes to indicate the impact of pathogen dependency on the country variable (used to describe the different household size distribution) in the scenario with 10 pathogens. The covariate used is the coefficient for the degree of household isolation (0 to describe the independent scenario and 1 to describe complete household isolation in case of the dependency). The confidence intervals show the variability in the slopes across different country variables indicating different household size distributions. For studying the degree of household isolation, we use the Generalized Additive Model (GAM). GAM’s are an extension of the generalized linear model (GLM) allowing for some kind of smoothing of the predictor variables. The advantage of GAMs is that it allows us to deal with highly non-linear and non-monotonic relationships between the response and the predictor variables often driven by the observed data at hand [26, 27]. GAMs are also used in this work to model the dependency of incidence proportion of infections stratified by household size where a non-linear relationship is observed.

## Results

**Fig. 3:**
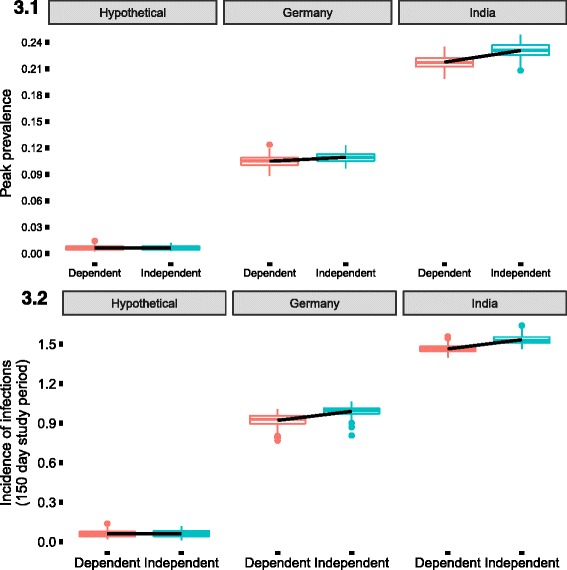
Difference across the country locations indicating the different household size distribution and the coefficient of household reduction during the symptomatic phase of the infection. The slope is obtained from the linear model to indicate the change caused by the most extreme difference in the coefficient due to the dependent scenario (all pathogens interacting with dependency) and the independent scenario (all pathogens working independently). This is also visible in Table 6. The outcomes of interest that have been presented are 3.1- peak prevalence during the observed epidemic, 3.2- incidence of infections during the 150 day period of interest. (Numbers above 1 indicate that cumulative probability of infections during the study period was above 100%)

In our simulations, pathogen 6 demonstrated behaviour similar to a pandemic influenza epidemic. Hence this was the most severe pathogen in our list. Pathogen 10 was the second most severe among the pathogens present. The differences in the household size distribution described through the country variable have been demonstrated in Table 5. Smaller household sizes were associated with less severe epidemics demonstrated through the smaller values of the peak prevalence as well as the lower incidence proportion. And the epidemic process was also slower which would be indicated through the delayed median time in reaching the peak prevalence of infections in the study period. The epidemic almost never occurred (low values of incidence and prevalence and high variability in time to reach the peak prevalence) in the hypothetical population cohort where within household infection transmissions were completely absent (Fig. 3). Simulation of pathogen 6 and pathogen 10 jointly was associated with household isolation during the symptomatic phase of an episode, and this brought in competition within the two pathogens during the epidemic process. We tested the hypothesis that incidence proportion and peak prevalence was higher in individual simulations of the pathogen as opposed to the joint interaction of the two pathogens in a system. The difference could be observed only where the epidemic occurred (i.e. not in the hypothetical population cohort). Furthermore we also evaluated the hypothesis that the sum of the independent peak prevalence and the incidence proportion from the two pathogens was greater than the joint overall peak prevalence and incidence proportion in the two pathogen system. Here too the difference was observed except in the hypothetical cohort (Table 5). In the two pathogen system the time taken to reach the peak prevalence was dominated by the pandemic pathogen (pathogen 6). However there was no difference observed in this duration in the two pathogen system and separately simulated individual pathogen systems. When two exactly same pathogens were considered then the results of the comparison were similar. However when two pathogens were more aggressive (characteristics of pathogen 6) then the difference in the peak prevalence of infections between jointly modelling them and considering them individually, were significantly higher than the scenario when two pathogens had moderate characteristics (characteristics of pathogen 10).

**Table 5:** Summary and comparison of two pathogen system (S2) vs. one pathogen system (S1). The pathogen is indicated in the parenthesis. S1(P6 + P10) indicates the sum of the individual values from the pathogen independently whereas S2(P6 + P10) indicates the system where the household isolation introduces pathogen dependency and the pathogens function jointly. The outcomes of peak prevalence and incidence proportion (during the 150 day period) along with their 95% confidence intervals (based on Monte-Carlo simulations) are shown in the summary section. The comparison section displays the non-parametric *p* values (based on the Mann-Whitney-Wilcoxon test) obtained when comparing the pathogen systems over the simulation runs

	Peak prevalence			Incidence proportion		
	Hypothetical	India	Germany	Hypothetical	India	Germany
S1 (P10)	0.0006 (0.0004,0.001)	0.078 (0.072,0.084)	0.019 (0.015,0.025)	0.005 (0.002,0.009)	0.617 (0.586,0.640)	0.318 (0.276,0.357)
S1 (P6)	0.006 (003,0.011)	0.179 (0.170,0.187)	0.096 (0.089,0.104)	0.061 (0.021,0.102)	0.763 (0.753,0.771)	0.654 (0.640,0.669)
S2 (P10)	0.0006 (0.0003,0.001)	0.070 (0.065,0.079)	0.014 (0.009,0.021)	0.004 (0.002,0.009)	0.589 (0.564,0.612)	0.279 (0.198,0.334)
S2 (P6)	0.006 (0.003,0.010)	0.169 (0.162,0.177)	0.094 (0.087,0.101)	0.055 (0.026,0.099)	0.761 (0.750,0.771)	0.652 (0.636,0.667)
S2 (P6 + P10)	0.006 (0.003,0.011)	0.211 (0.198,0.225)	0.105 (0.098,0.113)	0.061 (0.030,0.102)	1.351 (1.318,1.378)	0.931 (0.857,0.983)
S1 (P6 + P10)	0.007 (0.003,0.012)	0.258 (0.246,0.267)	0.116 (0.104,0.125)	0.068 (0.026,0.107)	1.383 (1.341,1.410)	0.970 (0.933,1.015)
*Comparison*						
S1(P10) vs. S2(P10)	0.590	<0.001	<0.001	0.543	<0.001	<0.001
S1(P6) vs. S2(P6)	0.471	<0.001	0.029	0.350	0.029	0.134
S1(P6 + P10) vs. S2(P6 + P10)	0.175	<0.001	<0.001	0.672	<0.001	<0.001

Since the multi pathogen scenario would be a more probable model, we consider 10 pathogens as described in Table 4. These include among them pathogen 6 and pathogen 10 which have been described before. We now vary the coefficient of household reduction process between the extremes of 0 and 1, indicating pathogens functioning independently in the population and pathogens interacting within the population respectively. Looking across the different country variables for varying household size distributions, we observed that here too societies with larger household sizes had an accelerated and more severe epidemic (Fig. 3 (3.1, 3.2) and Additional file 1). Also we looked at the difference introduced by the extreme values of household isolation during the infectious phase using the slope of the linear regression model. The summary of the slopes indicated the differences when the epidemic took place (Table 6). For the German household size and hypothetical household size distributed cohort we could not observe any significant decrease in the speed of the epidemic as opposed to the cohort with the Indian household size distribution where there was an accelerated epidemic observed with an increased coefficient of household isolation.

**Table 6:** Comparison of slopes across the different country locations. This indicates the observed difference in the outcomes from the epidemics due to the differences in the coefficient of household isolation (the extreme scenarios of complete dependency versus pathogens functioning independently) and the household size distribution in the country location used as shown in Fig. 3 (3.1, 3.2)

	Hypothetical	India	Germany
Peak Prevalence	(−0.0006,0.0008)	(−0.015,-0.011)	(−0.006,-0.003)
Time to reach peak prevalence	(−22.542, 4.102)	(1.252, 2.427)	(−0.932,1.732)
Incidence proportion	(−0.008,0.006)	(−0.079, −0.060)	(−0.081,-0.056)

**Fig. 4:**
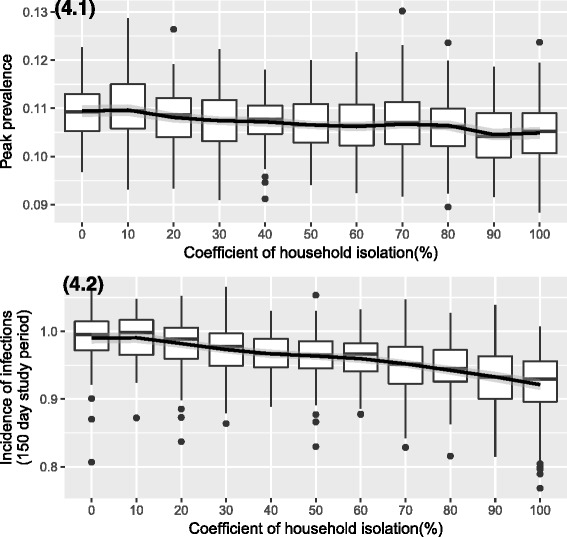
Epidemic outcomes with varying degree of household isolation. We observe a decrease in peak prevalence (4.1), and incidence of infections (numbers above 1 indicate that cumulative probability of infections during the study period was above 100%) (4.2), with the increase in the degree of household isolation during the infectious phase

We analysed the impact of changing the coefficient of household isolation during the infectious period by varying it from 0 to 100% in steps of 10% for the cohort with the German household size distribution. The time taken to reach the epidemic peak remained unchanged with the variation of the household isolation coefficient. However there was a decrease in the epidemic peak and the incidence proportion of infections with an increase in household isolation coefficient. This would be a result of decreasing contacts during the infectious phase with the society making individuals less vulnerable to newer infections during this period. The results are represented in Fig. 4 (4.1 and 4.2) and the smoothed coefficients based on the coefficient of household isolation for the GAM regression was highly significant for the outcomes of incidence proportion and peak prevalence of infections (both below 0.0001). However it was not significant for the outcome of the time taken to reach the peak prevalence. Fitting of a simple linear regression model with the above scenario also gave us similar results and the slope was always negative (except for the outcome of time to reach the incidence peak). However the fit was better with the GAM model through the median points.

**Fig. 5:**
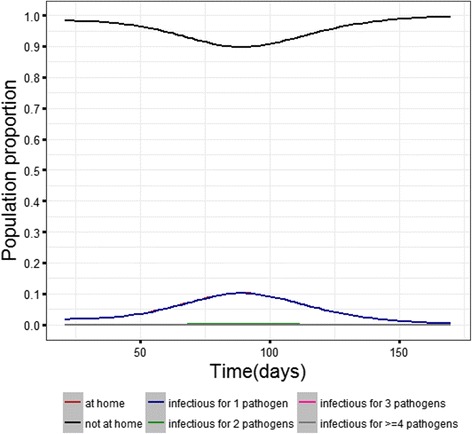
Simulation results showing the average population proportion from 100 simulated epidemics during the epidemic period that are under household isolation for being symptomatic for infections. The black and the red line indicate how the proportion of people acquires infections during the course of the epidemic and then recover with time. The red line shows that a maximum of a tenth of the population remains at home on an average during the epidemic period. The blue line almost covers the red line indicating that majority of the infection episodes are caused by one pathogen. The pink and the grey lines are almost close to zero at all the time points indicating how unlikely it is for an individual to be infected with more than one pathogen at a time

Additionally, we looked at the proportion of individuals who were at home at a given time point. Together with this we also looked at the distribution of the proportion of people who were infectious for one or more pathogens on a given time point. We assessed these proportions in simulations for the German household size distribution, with immune individuals in the population, and 10 pathogens interacting with each other during the epidemic process (Fig. 5). Our calculations showed that majority of the cases where the person was symptomatic and remained at home, was due to one pathogen with a proportion of 0.9959 (0.99, 1.00) (median with 5th and 95th percentile values in bracket). For two pathogens at a time, the proportion was 0.003 (0.00, 0.01) (median and with 5th and 95th percentile values in bracket) whereas for three pathogens at a time the median proportion was already zero. This could also be seen in Fig. 5, where the proportion of infectious individuals for two or more pathogens at a time point was very close to the zero line. For the similar scenario with the household size distribution of India in the cohort, we obtained similar results. The proportion of household stays due to one pathogen infection was dominant, 0.9963(0.96, 1.00) (median with 5th and 95th percentile values in bracket). There were also some rare cases of being simultaneously infected by three or four pathogens.

**Fig. 6:**
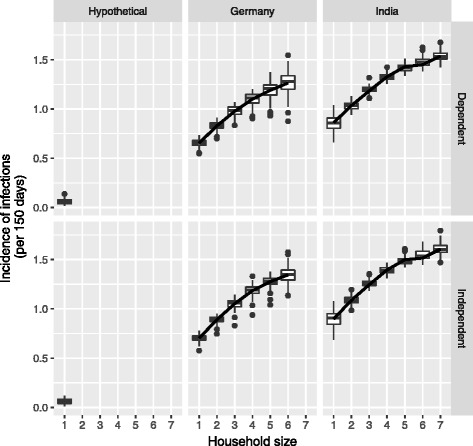
Incidence of infections stratified by household size (numbers above 1 indicate that cumulative probability of infections during the study period was above 100%)

We also analysed the incidence proportion stratified for the household size in the different population distributions. We saw an increase in incidence proportion of infections with increase in household size (Fig. 6). Here also we used a GAM model to represent the nonlinear relationship between incidence proportion and household size. For the hypothetical population cohort we could not have any relationship because it only represented one membered household. The incidences of the one member household were also different across the different population distributions with higher incidences in population with larger household sizes.

**Fig. 7:**
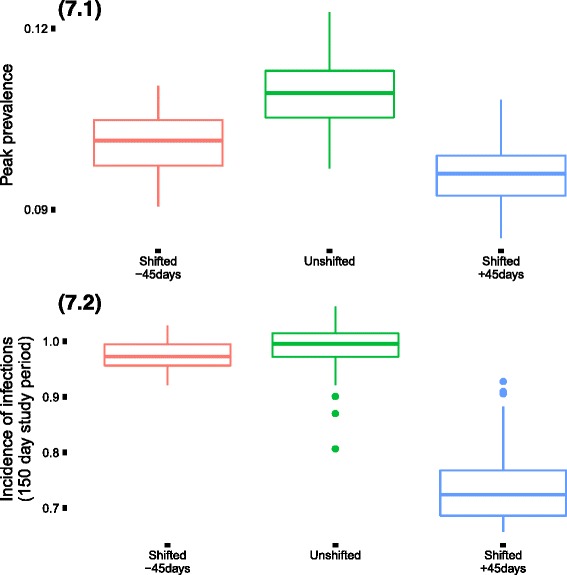
Epidemic outcomes for the base case scenario (all pathogens temporally aligned in their seasonality) in comparison to the scenarios where pathogen 10 has a shifted temporal trend. The shifting reduces the intensity of the epidemic. The reduction is more when there is a delayed peak in the epidemic for pathogen 10 as opposed to an earlier peak. (Incidence numbers above 1 indicate that cumulative probability of infections during the study period was above 100%)

Finally, we observed the impact of shifting the temporal trend for pathogen 10 (representing the characteristics of RSV virus) as opposed to pathogen 6 (representing the characteristics of pandemic influenza virus) and the remaining 8 viruses in the 10 pathogen system. The trend for pathogen 10 was shifted by using the different values for *t*_0_ as +45 and −45 days. We performed the simulations only for the German population type with dependency among pathogens (complete household isolation during infectious phase). In comparison to the base case scenario, there was a decrease in the peak prevalence as well as the incidence proportion due to the temporal shift in the trend for pathogen 10. The decrease was significantly higher for the shift where the peak for pathogen 10 is delayed by 45 days as opposed to the peak coming forward by 45 days. The time taken to reach the epidemic peak remained unchanged (Fig. 7 (7.1 and 7.2)). Also the decrease in the incidence proportion was comparatively smaller than for the peak prevalence between the scenario where the peak was forward by 45 days for pathogen 10 and base case.

## Discussion

We have proposed an agent based model to study the behaviour of epidemics under the influence of multiple pathogens working simultaneously in the population. With the presence of two pathogens in such a system without the influence of any other effect, we could demonstrate how the interference of the pathogens in the infection process played a role in controlling the epidemic process (lower number of infected individuals as well as lower daily incidence proportion). The interference among pathogens was introduced through the assumption of household isolation during the period of being symptomatically infectious, where the individual was immune to the risk of acquiring infections from outside the household. To our knowledge this was the first time for studying the behaviour of an epidemic process incorporating the influence of multiple pathogens using an agent based model. We further went on to present a more general scenario where there are 10 pathogens, and also the impact from recovered individuals being present in the population at the start of the epidemic process.

Our simulations were performed to study the impact of the dependency among pathogens as opposed to pathogens functioning independently (2 extreme levels for the coefficient of household isolation during the infectious period), and the household size distributions of different populations (three different populations with varying household size distributions) The population system reached a stable state at the end of simulation period, confirming that the epidemic had almost died out in 150 days (approximately 5 months during winter season). The dependencies among pathogens were important determinants in controlling the epidemic process. Additionally, the household size distributions did produce significant differences in the peak of the epidemic (peak prevalence) and the incidence proportion in the study period of interest. For common respiratory infections like influenza and common cold, household size can be an important factor determining their spread as seen for influenza or influenza like illnesses based hospitalizations: the population structure difference has accounted for a third of the observed variation [28]. In our simulations we observed that household size distribution influences the speed of the epidemic. Population with larger household sizes reached the peak of the epidemic much faster than those with smaller household sizes. Looking at the incidence of infections across household sizes, we could see that larger households were associated with more infections due to the intra household infection spread, consistently with assumed random mixing within the household.

Looking across the different pathogens, we observed that the infectious period also is important in shaping the severity of the epidemic. Pathogen 6 and 10 as considered in the simulation have almost similar characteristics except for the duration of the infectious phase, but this resulted in different severity of the epidemic. Also in the multipathogen scenario, the epidemic characteristics are dominated by pathogen 10. Shifting of the temporality to introduce a peak 45 days before for pathogen 10 allows for more infections in the multipathogen system as opposed to a delay in the peak.

Our simulation study does come with limitations. There are common challenges associated with agent based models, especially in statistical methods for hypothesis testing in combination with determining the number of appropriate simulation runs [29]. In addition to the standard challenges, our assumptions are largely simplistic in nature, assuming for random mixing within the household and the population is looked upon as an assortment of homogenous agents. The increase in contacts with the increasing household size may not necessarily take place. Secondly, we induce a sort of isolation for the transmission process of infections, but we do not account for the specific severity of the infections, except for the duration of being symptomatic and infectious. The severity of the pathogen can directly influence the duration of isolation. Even for our sensitivity analysis, we assume this parameter to be same for all the pathogens. Additionally, we also assume same transmissibility characteristics for all the pathogens. These are strong assumptions that have been made for the realization of the system in a simplistic way. However, this model can be extended easily to observe more complex realizations of a realistic system.

## Conclusion

Through our agent based model formulation, we could demonstrate the importance of considering the multi-pathogen interactions in controlling the spread of infections during an epidemic process. Household size and dependency among pathogens are important factors in determining the outcome of the epidemic. Future prospective studies in household cohorts looking at pathogen identification and coinfections can provide quantitative measures for specific characteristics of the multi-pathogen system. This kind of data can also be used to test the validity of the assumptions made in simulation models.

## Additional file

Additional file 1: Time taken to reach the peak prevalence varies according to the household size distribution in the cohort. Populations with larger households on an average experienced the epidemics at an accelerated rate compared to populations with smaller households on an average. (PNG 24 kb)
