# Supplementary figures and images for: Effects of pathogen dependency in a multi-pathogen infectious disease system including population level heterogeneity – a simulation study
Source: Theor Biol Med Model. 2017 Dec 13;14:26. doi: 10.1186/s12976-017-0072-7 (PMC5729270; doi:10.1186/s12976-017-0072-7)

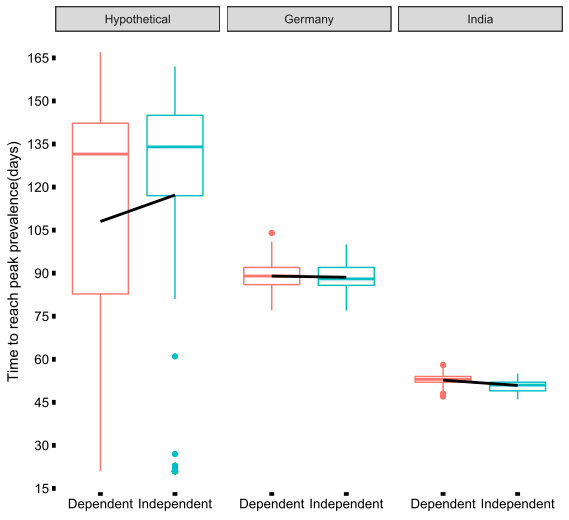

Supplement: Additional file 1: — Time taken to reach the peak prevalence varies according to the household size distribution in the cohort. Populations with larger households on an average experienced the epidemics at an accelerated rate compared to populations with smaller households on an average. (PNG 24 kb) [file 12976_2017_72_MOESM1_ESM.png]
